# Cold Stress Response Mechanisms in Anther Development

**DOI:** 10.3390/ijms24010030

**Published:** 2022-12-20

**Authors:** Borong Huang, Yubo Fan, Lijiao Cui, Cheng Li, Changkui Guo

**Affiliations:** Collaborative Innovation Center for Efficient and Green Production of Agriculture in Mountainous Areas of Zhejiang Province, College of Horticulture Science, Zhejiang Agriculture and Forestry University, Hangzhou 311300, China

**Keywords:** cold stress, anther development, tapetum, sugar metabolism, phytohormone

## Abstract

Unlike animals that can escape threats, plants must endure and adapt to biotic and abiotic stresses in their surroundings. One such condition, cold stress, impairs the normal growth and development of plants, in which most phases of reproductive development are particularly susceptible to external low temperature. Exposed to uncomfortably low temperature at the reproductive stage, meiosis, tapetal programmed cell death (PCD), pollen viability, and fertilization are disrupted, resulting in plant sterility. Of them, cold-induced tapetal dysfunction is the main cause of pollen sterility by blocking nutrition supplements for microspore development and altering their timely PCD. Further evidence has indicated that the homeostatic imbalances of hormones, including abscisic acid (ABA) and gibberellic acid (GA), and sugars have occurred in the cold-treated anthers. Among them, cold stress gives rise to the accumulation of ABA and the decrease of active GA in anthers to affect tapetal development and represses the transport of sugar to microspores. Therefore, plants have evolved lots of mechanisms to alleviate the damage of external cold stress to reproductive development by mainly regulating phytohormone levels and sugar metabolism. Herein, we discuss the physiological and metabolic effects of low temperature on male reproductive development and the underlying mechanisms from the perspective of molecular biology. A deep understanding of cold stress response mechanisms in anther development will provide noteworthy references for cold-tolerant crop breeding and crop production under cold stress.

## 1. Introduction

Plants with a sessile lifestyle tolerate biotic and abiotic stresses around them by adapting to the change of external environments through self-regulation. One such stress, cold stress, as a negative regulator, limits the distribution and yield of plants [1,2,3]. When the temperature is lower than the threshold that plants can endure, plants must speedily adjust their internal physiology and biochemistry for maintaining homeostasis. In turn, plant growth and development are negatively impacted [4].

Considering the increase of extreme weather in the future, it is very important to enhance the ability of crops to resist abiotic stresses for food security. Extreme low temperatures have a detrimental effect on reproductive organs, which will reduce the yield of several species [5]. Of them, anther is one of the most sensitive organs to cold stress. The abrupt decrease in temperature affects pollen viability and fertility, anther wall formation, anther dehiscence, pollen tube growth, and fertilization, ultimately leading to varying degrees of sterility [6,7]. Cold hinders the supplement of nutrients to microspores via tapetal cells, which might be the main cause of pollen infertility [8]. Further, the infertility of pollen under cold results in the reduction of plant reproduction and crop productivity. Therefore, it is significant to clarify the cold responsive mechanisms underlying cold-related male sterility.

As reported, pollen development includes two main stages [9]. The first one is after anther morphogenesis and microspore mother cells (MMCs) meiosis to form a tetrad; the surrounding callose is degraded for releasing microspores. Germ cells and somatic cells jointly promote the beginning of meiosis. The second stage is that microspores undergo mitosis twice to form pollen. Meeting low temperature at these stages will lead to anther distortion and abnormal pollen grain development, and even male sterility [10]. Pollen fertility is associated with sugar transport by tapetal cells. Tapeta enclose MMCs and provide various substances needed during the meiosis of MMCs and their subsequent development. Studies have demonstrated that the untimely depletion of tapetum leads to the abnormal apoptosis of microspores, indicating that tapetum plays an indispensable role in male reproductive development [9]. Herein, we discussed the cold response mechanism in pollen development from two perspectives including the physiology and the molecular mechanism.

## 2. Effects of Cold Stress on Pollen and Anther Development

Upon sensing low temperature, cell membrane receptors transmit signals into cells, which regulate physiological metabolism and molecular levels, ultimately allowing plants to adapt to stress and build tolerance. Low temperature reduces cell membrane fluidity and alters membrane protein conformation [11,12,13]; thereafter, the reactive oxygen species (ROS) are bursting with induced membrane lipid peroxidation and elevated malondialdehyde levels, and then the cell membrane structures are disrupted [14]. In addition, a high percentage of unsaturated fatty acids facilitates the cold tolerance of the plasma membrane. When the temperature drops below 0 °C, ice crystals are formed at the plasma ectodomain, which induce dehydration inside the cell, and eventually damage the membrane system [15]. During the reproductive stage, the numbers of proline and polyamines are overproduced in response to cold stress [16,17], for further alleviating enzyme denaturation and inactivation. Moreover, the transcript levels of various polyamines synthesis genes, such as arginine decarboxylase (*ADC*), spermidine synthase1-2 (*SPDS1-2*) and spermine synthase (*SPMS*), are fast induced against the harsh condition [17].

**Figure 1:**
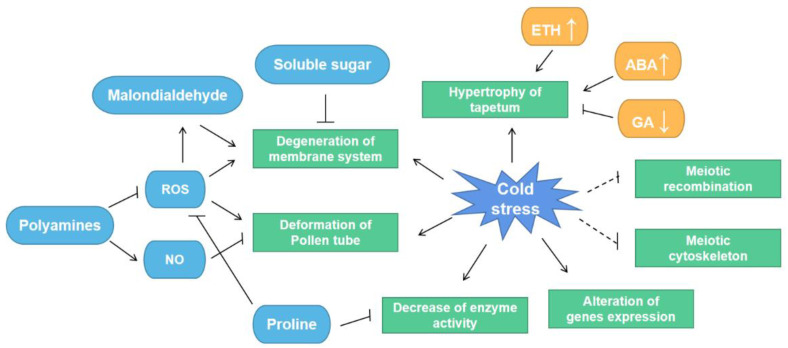
A generalized map of the effects of different regulators on anther development under cold stress. Cold stress alters gene expression and reduces enzyme activity. Low temperature induces ROS accumulation, and thereby disrupts cell membrane structure and pollen tube elongation. Polyamines and proline respond to the persecution of reproductive organs by cold stress by reducing ROS. The mechanisms of meiotic reorganization and cytoskeleton disruption in anthers under low temperature need to be further investigated.

The process of male gametophyte formation is sensitive to low temperature. After the cold treatment of Arabidopsis pollen at 4 °C for 48 h, only 43.4% of pollen grains survived and 39.2% of pollen was germinated [18]. Cold-tolerant crops produce more viable pollen [16]. The normal anther wall consists of epidermis, endothecium, middle layer, and tapetum. Cold stress affects the cytological aspects of pollen development. After 48 h of cold treatment, the cytoplasm of the cytoplasm of the tapetal cells of both the cold-tolerant rice ‘Longjing25′ and the cold-sensitive ‘Longjing11′ is condensed. Differently, ‘Longjing11′ stays at the pollen mother cell stage, whereas ‘Longjing25′ enters meiosis with the decreased middle layer cells [19]. Low temperature increases the rate of meiotic recombination by enhancing the crossover of homologous chromosomes [20,21]. Exposure to cold stress during male meiosis disrupts the formation and function of the radial microtubule arrays (RMA) and is accompanied by alterations in callose deposition and defects in the cell plate, and culminating in the formation of binuclear and polynuclear microspores [22]. These microspores fuse prior to pollen meiosis I (PM I) to produce a second division restitution (SDR) type 2n pollen. Transcriptomic analysis indicates that cold stress affects specific actin dynamics-related genes and regulators, resulting in cell plate defects [23]. Low temperature-induced meiotic recombination and cytoskeletal changes facilitate genome diversity and species evolution. Cold stress interferes with pollen tube elongation during anther development. The cold treatment of *Arabidopsis* at 4 °C for 24 and 48 h reduces pollen tube elongation rate to 60.5% and 33.8%, respectively [18]. Normally, pollen tubes are cylindrical with a dome at the apex, whereas swelling and curling are occurred at the apex under cold stress [24]. Low temperature disrupts the actin cytoskeleton of pollen tubes, leading to callose deposition at the apex of the pollen tube cell wall. Cold stress also alters the distribution of Ca^2+^ and ROS in pollen tubes [25]. Nitric oxide and polyamines mitigate the interference of cold stress on pollen tube elongation, and polyamines regulate pollen tube growth by modulating the nitric oxide signaling pathway and ROS levels [26]. These regulators synergistically affect anther development under low temperature (Figure 1).

## 3. Cold Stress Interferes with Tapetal Development and Programmed Cell Death (PCD)

In addition to transporting nutrients for microspore development, tapetum also provides sporopollenin precursors to help form pollen exine. MYB80/MYB103/MALE STERILE 188 (MS188) and ABORTED MICROSPORES (AMS) are tapetal-cell-specific transcription factors, which activate *CYP703A2* after interaction, to promote the sporopollenin biosynthesis of tapetum [27]. After PCD, the tapetum releases elaioplasts and tapetosomes, which temporarily store lipids in the cells. These subcellular organelle contents are deposited to form the pollen coating [28]. In conclusion, tapetal development and PCD affect pollen fertility [29]. Some transcription factors related to tapetal development have been identified and confirmed. *MALE STERILE1* (*MS1*) is the key gene for delaying tapetal PCD. During the microspore release stage, the *MS1* gene is mainly expressed in tapetum. In *ms1* mutant, tapetal vacuolation and abnormal PCD results in the inability to form functional pollen exine and ultimately microspore disintegration [30,31]. In *Arabidopsis*, these genes together constitute a regulatory network affecting tapetal development and PCD [32]. 

Under cold stress, the tapetum continues to expand until the pollen mature stage, which is different from the early degradation of tapetum caused by heat stress [33,34]. Staining rice anthers with tetrazolium under cold stress indicates that most pollen grains are inactive at 20 °C, and mature anthers have no pollen grains at 16 °C [35]. Oda et al. analyze the morphological characteristics of anther development under low temperature stress using low tolerance rice ‘Sasanishiki’, and find that tapetal degradation does not only occur during the mononuclear microspore stage, but also abnormally expands, resulting in blocked pollen development and decreased vitality, suggesting that cold stress causes pollen sterility by damaging the PCD of tapetum [34]. Through the study of pollen ultrastructure, it is found that cold stress changes the normal development of microspores and surrounding cell layers. In the cold-induced tetrad, callose is degraded prematurely, microspore wall forms abnormally, and tapeta are hypertrophy and vacuolization. Excessive starch accumulation in chloroplasts is accompanied by thylakoid and membrane degeneration [36]. Through the investigation of thermophilic crop watermelon, it is found that cold stress does not affect anther morphology, but delays tapetal PCD and promotes sporophytic tissues PCD, resulting in the inability of pollen to crack, thus reducing fertility. Further studies show that the expression of *ClMYB80*, *TAPETUM DEGENERATION RETARDATION* (*ClTDR*), *ETERNAL TAPETUM 1* (*ClETA1*), *DEFECTIVE IN TAPETAL DEVELOPMENT AND FUNCTION 1* (*ClTDF1*), *ClDYT1b*, and *ClMS1a* genes is decreased in watermelon pollen under cold stress. These results demonstrate that cold hinders PCD in tapetum by reducing the expression of PCD-related genes [37]. PHYTOCHROME-INTERACTING FACTOR 4 (PIF4) integrates many pathways such as light, temperature and hormones to regulate plant adaptation to environmental stress [38]. *PIF4* is mainly expressed in tapetum and microspores. Under low temperature stress, *slpif4* anthers produce more active pollen and higher fruit setting rate than wild type (WT). The overexpression of *SlPIF4* results in delayed tapetal PCD. The expression of PCD-associated genes in *SlPIF4*-overexpressing anthers is significantly higher than that in WT. These results suggest that SlPIF4 negatively modulates the cold tolerance of pollen development by regulating the gene regulatory network of tapetal development and PCD [39].

Cold stress influences the function of subcellular organelles in the tapetum. The failure of tapetal function may be related to the structural change of endoplasmic reticulum (ER) by observing the anther induced by low temperature. The ER synthesizes flavonoids, which are transported to the pollen after the degradation of the tapetum [40,41]. ER mediates the post-translational processing of peptides, and treatment of misfolded proteins by ER-associated degradation (ERAD) machinery [42]. At low temperature, the cytoplasmic components, and organelles of tapetal cells remain unchanged, but ER bodies are formed in ER. ER structures are no longer stacked normally, but form linear, wavy, annular, or circular shapes [35]. These abnormal structures are unable to maintain the normal function of tapeta, resulting in tapetum hypertrophy and microspore abortion. Some sporopollenin biosynthetic enzymes also exist in the ER involved in the synthesis of the pollen exine [43]. In addition, the ER participates in callase (β-1,3-glucanase) synthesis and transports it from tapeta to tetrad by synthetic vesicles [44]. Anther-specific protein 6 (A6), a callase, functions in degrading the callose around the tetrads, and AMS affects microspore release by regulating the expression of *A6* [27]. The β-(1,3)-galactosyltransferase UPEX1 is recently reported to synthesize and function in the tapetum to catalyze the glycosylation of A6. AMS affects A6 secretion within the tapetum by directly regulating *UPEX1* [45].

Low temperature affects the chloroplast membrane system and decreases the chlorophyll contents [46,47]. The degeneration of thylakoids, grana, and the outer membrane of chloroplast is observed in cold-treated anthers, accompanied by starch accumulation [36]. Pheophorbide an oxygenase (PaO) is a key enzyme that degrades chlorophyll. The *PaO* gene is also expressed in reproductive tissues. After low temperature treatment, *TaPaO1* shows increased expression during meiosis and is specifically expressed around the chloroplast. *RD29A-TaPaO1* transgenic tobacco line has no pollen under cold stress. These results reveal that chlorophyll is involved in low temperature induced pollen sterility [48]. *ZmGPAT6/ZmMs33* encodes a glycerol-3-phosphate acyltransferase (GPAT), which mediates lipid synthesis in the tapetum. The *zmgpat6* leads to the vacuolation and the PCD of tapetum in advance. Chloroplasts in maize anthers are located in endothecium, which provides nutrients and energy for anther development. The absence of major components of chloroplast membranes due to loss of *ZmGPAT6* function impairs chloroplast photosynthesis progression and starch transport, rendering pollen nutrient deficiencies ultimately abortion [49].

The changes of cellular redox status (especially ROS) affect tapetal PCD and reproductive development [50]. Mitochondria are major sources of ROS and are critical for PCD of cells [51]. Low temperature induces mitochondrial swelling and decrease, and membrane lipid phase transition [52]. Steiner et al. explore the effects of cold on subcellular organelles at different evolutionary levels in the ultrastructural level [53]. It is found that mitochondria are spherical and distributed separately at normal temperature. Under 4 °C cold stress, mitochondria begin to elongate, aggregate, and be partially fused. Under −2 °C freezing stress, mitochondria elongate and aggregate into local networks with outer membrane fusion; and mitochondria are also fused with mucilage vesicles. In addition, the ER and chloroplasts are swollen, as well as peroxisomes protruding into the mucilage vesicles. *UNDEAD* encodes an A1 aspartic protease that hydrolyzes proteins within mitochondria involved in PCD by altering mitochondrial membrane permeability. MYB80 mediates tapetal PCD by regulating UNDEAD [54]. Mitochondrial ATP synthase is inhibited in low temperature environment, which limits respiration and energy synthesis, and finally leads to infertility [55]. Further studies are needed to explore whether low temperature causes male infertility by affecting gene expression and enzyme activity in these subcellular organelles.

## 4. ABA Induces Pollen Sterility under Cold Stress

Under abiotic stress, ABA accumulation improves plant stress resistance, but hinders reproductive development [56,57]. For example, temperature stress leads to microspore invagination, abnormal tapetum degeneration and fertilization failure in *Arabidopsis* [58]. ABA biosynthesis mainly involves the zeaxanthin epoxidase (ZEP) and 9-cis-epoxycarotenoid dioxygenase (NCED), whereas ABA inactivation mainly relies on the C-8′ hydroxylation pathway [59]. The overexpression of *NCED* or *ZEP* in plants increases the amount of ABA biosynthesis and thereby improves stress tolerance [60]. At low temperatures, ABA levels in rice and chickpea anthers are abnormally elevated; and the exogenous application of ABA increases cold tolerance, suggesting that ABA is involved in cold induced pollen sterility [61,62]. However, whether cold treated or not, the content of ABA in cold-resistant variety ‘R31′ is lower than that in cold-sensitive rice variety ‘Doongara’ [62]. Therefore, it is speculated that plant cold tolerance is related to its ability to regulate the change of ABA level in low temperature environment. 

After cold stress, changes in endogenous ABA levels result from the co-regulation of ABA biosynthesis and catabolism [58]. The expressions of *OsNCED1*, *OsNCED3,* and *OsZEP1* are up-regulated in cold-sensitive rice under cold condition, whereas *OsNCED3* expression is unchanged and *OsZEP1* expression is decreased in cold-tolerant rice. Under normal conditions, the expression of ABA 8’-hydroxylase genes in cold-tolerant variety is higher than that in a cold-sensitive variety. However, the expressions of *OsABA8OX2* and *OsABA8OX3* in cold-tolerant variety are inhibited after cold treatment, whereas their expressions are lower than that in cold-sensitive variety [62]. The study has shown that GUS staining mainly appears around the vascular bundle by using *OsNCED3*-GUS transgenic rice anthers, indicating that ABA is synthesized in the anther vascular bundle. On the other hand, the tapetum specific strong promoter *OsG6B* is used to promote the expression of *TaABA8′OH1* in rice, and the results indicate that the accumulation of ABA in transgenic lines is decreased under low temperature environment, thus improving the cold resistance of rice [63]. The above results demonstrate that keeping low levels of ABA by balancing ABA biosynthetic and catabolic pathways is beneficial for plants to resist external cold stress.

ABA impedes tapetal PCD [64]. ABA accumulates during early anther development and promotes pollen mother cell and tapetal separation. After that, the ABA levels begin to decrease at the tetrad stage and reach the lowest levels when the tapeta are completely degraded [65]. N6-methyladenosine (m^6^A) is methylation of the N6 position of adenosine [66]. It has been reported that m^6^A induces pollen sterility by co-regulating ABA level and tapetal PCD under moderating low-temperature (MLT) stress [67]. MLT treatment delays the PCD of tapetum and changes the deposition of pollen exine, resulting in tomato pollen sterility. The changes of m^6^A level and related genes expression level hint the importance of N6-methyladenosine in pollen in the face of moderate cold stress. An analysis of transcripts at normal temperature and MLT reveals a subset of transcripts with significantly altered m^6^A methylation levels, such as lipid metabolism, carbohydrate derivative binding, and ATP binding pathways. The increased methylation levels of *ATP-binding cassette G31* (*SlABCG31*) involved in ABA transport in MTL stress cause significantly higher ABA levels, indicating that ABA participates in the regulation of pollen development under MLT stress. In summary, m^6^A methylation modulates tapetal and microspore development in part through the ABA signaling pathway to cause pollen sterility at low temperatures [67]. Furthermore, it has been reported that ABA regulates tapetal PCD by acting together with other phytohormones [64].

## 5. GA Regulates Pollen Development and Tapetal PCD under Cold Stress

Plants continuously receive endogenous and exogenous signals during their growth and development. During the ‘Green Revolution’, dwarf genes are introduced into rice and wheat to cultivate lodging resistant and high-yield food crops. It is subsequently discovered that the ‘Green Revolution’ is associated with gibberellins (GAs). *Semi-dwarf 1* (*SD1*) and *Reduced height 1* (*Rht1*) function in regulating GAs biosynthesis and signaling pathways, respectively [68]. GAs act synergistically in plants’ life activities and other factors [69]. The GA pathway regulates plant flowering by integrating environmental signals and other phytohormones [70,71]. GAs also participate in plant resistance to stress, and play an antagonistic role with ABA, in controlling anther development [72]. The generation of bioactive GA from *trans*-geranylgeranyl diphosphate (GGPP) requires the catalysis of a variety of enzymes, among which important enzymes are GA20-oxidase (GA20ox) and GA3ox [73]. An incomplete development of stamens and anthers in the *ga20ox1 ga20ox2* double mutant results in male abortion [74]. After being catalyzed by GA2ox, active GAs are converted to inactive Gas, while AtGA2ox contributes to *Arabidopsis* reproductive development and stress resistance [69]. The maintenance of GA homeostasis is required for life activities of plants to proceed normally. GIBBERELLIN INSENSITIVE DWARF1 (GID1) is a receptor for bioactive GAs in the GA signaling pathway [75]. There are three homologous GID1 receptors in *Arabidopsis*, and either double or triple mutants exhibited obvious dwarf and male sterility phenotypes [76]. When the C-terminal of GID1 binds to GAs, it results in a structural change of its N-terminal, forming the GA-GID1-DELLA complex, which is degraded by ubiquitination to relieve DELLA inhibition of plant growth [77,78]. As the major negative regulator of the GA pathway, DELLA can also combine with some growth regulators to cope with cold environment [79]. GA signaling pathway regulates tapetum development and pollen fertility through GA-regulated MYB (GAMYB) [80,81]. *Swollen anther wall 1* (*SAW1*) is also involved in the mechanism by which GA regulates pollen development. The tapetum in *saw1* rice anthers is delayed in PCD and metabolically active within the organelles. SAW1 changes GA content by regulating *OsGA20ox3*, which is specifically expressed in anthers, and then affects *GAMYB* gene expression [82]. GA, via DELLA protein SLR1 in rice, interacts with tapetum-development-related transcription factors UNDEVELOPED TAPETUM1 (UDT1), TDR to prevent tapetal PCD [83]. These results suggest that both biosynthetic and signaling pathways of GA affect plant male reproductive development and that there is a feedback effect between these two pathways.

Reduced GA level inhibits plant growth thereby improving its survival and fitness in cold stress. Low temperature decreases pollen number and viability, and assays of its endogenous hormones exhibit a decrease in bioactive GA in anthers [84]. GA2ox, which makes GAs inactive, is not detective in pollen [34], whereas GA biosynthetic genes *GA20ox* and *GA3ox* are continuously transcribed with pollen development and are most highly expressed at the binuclear pollen stage. Cold inhibits the expression of biosynthesis genes through OsWRKY53, resulting in the overall decrease of endogenous GA content [83,85]. The CBF pathway is a crucial way to adapt to cold stress. Low temperature induces the transcripts of *CBF* genes, whereas overexpressing *CBF* genes makes *Arabidopsis* sacrifice male fertility and plant height to improve cold tolerance [85]. CBFs under low temperature conditions function in reducing GA content and promoting DELLA protein accumulation by elevating *GA2ox* genes, thereby partially facilitating plant adaptation to cold stress [85,86]. An increased expression of the *SlPIF4* gene enhances cold tolerance in tomato under low temperature and far-red light conditions. Probably, SlPIF4 activates both *CBF* and *GA-INSENSITIVE 4 (SlGAI4)* expressions. On the other hand, it elevates ABA and inhibits GA by regulating hormone synthesis and degradation genes. Ultimately, PIF4 increases tomato cold tolerance by combining light, temperature, and hormonal signals [87,88]. In addition, GA regulates other hormone levels to maintain the homeostasis of anthers under low temperature conditions [84,89]. 

## 6. Sugar Metabolism and Transport Affect Pollen Development

Sugars exert energy providing and signal transducing functions in pollen development [90]. Anthers cannot carry out photosynthesis and need to transport sugars from the source cells for their development. Carbohydrates are transported from the anther epidermis to the middle layer via the symplastic pathway and subsequently sucrose is transported to the tapetum and developing microspore cells via the apoplastic pathway [91], mediated by invertases and sugar transporters [92]. Sugar transporters mainly include monosaccharide transporters (MST) and sucrose transporters (SUT) that consume energy, as well as the sugar will eventually be exported transporters (SWEET) that transport sugar along the concentration gradient [93,94]. The AtSWEETs in *Arabidopsis* are divided into four phylogenetic clades and regulate sugar transport and distribution [95]. Functional analysis of this family shows that AtSWEET1-8 included in clade II are hexose transporters, while clade III composed of AtSWEET9-15 and clade IV composed of AtSWEET16-17 transport sucrose and fructose, respectively [95]. In the synergistic action of these sugar transporters to transfer diverse sugars into the tapetum, and the form of sugars varies according to the subcellular in which they are located [96]. Sucrose in the tapetum, on the one hand, is hydrolyzed to glucose and fructose by invertase (INV), and then glucose-6-phosphate (G6P) is formed under the action of hexokinase. The other is hydrolyzed by sucrose synthase (SuSy) to fructose and UDP-glucose, where it further forms glucose-1-phosphate (G1P) and G6P. G1P and G6P synthesize starch in amyloplasts. Hexoses in chloroplasts and mitochondria regulate tapetal PCD through ROS signaling pathways. Sucrose and hexose are transported from tapetum to developing pollen grains through SUT and SWEET transporters to synthesize starch [93,94]. As one of the indicators of pollen ripening, starch begins to accumulate in microspores after PM I, and reaches a maximum at the binuclear stage [97]. Subsequently, starch starts to degrade into soluble sugars such as sucrose and its hydrolysates glucose and maltose [98]. The timely synthesis and degradation of callose are conducive to tetrad formation and microspore release. Therefore, abiotic stress hinders sugar supply leading to pollen sterility [99].

Low temperature destroys the apoplast pathway to transport carbohydrates to the tapetum and microspores, resulting in tapetum hypertrophy and pollen malnutrition, which eventually leads to infertility. Cold stress renders pollen unable to proceed to PM I, resulting in reduced starch deposition in pollen grains and increased sucrose accumulation in anthers. Starch deficiency leads to failure of pollen and pollen tube germination. Sucrose accumulation in anthers is presumed to be due to a significant reduction in invertase activity. Through the identification of invertase genes, the expression of *OsINV4* is found to be specifical in the tapetum, and its expression disappeared as the tapetum degraded, indicating that OsINV4 is anther specific cell wall invertase [100]. To further characterize the effects of cold stress on the apoplastic pathway, anther monosaccharide transporter genes, *OsMST7* and *OsMST8*, are identified [62]. The expression patterns of *OsMST8* and *OsINV4* in the tapetum are consistent, whereas *OsMST7* is expressed in the anther wall and pollen. Cold stress suppresses gene expression, and sugars are not transported to the tapetum but stored in the anther wall in the form of starch [101]. Low temperature also causes the accumulation of sucrose within young ears of cold sensitive wheat, and the increase of sucrose content is higher in cold tolerant cultivars. An analysis of sucrose metabolism related enzymes reveals that the activities of SUS and INV enzymes related to sucrose degradation are fluctuated and reduced with decreasing temperature, respectively. An analysis of sucrose metabolism related genes in wheat ears after one day of cold treatment shows that the two genes encoding SUS are differentially expressed: *TaSUS1* expression is increased, whereas *TaSUS2* expression is decreased. Cold stress reduces sucrose transport with the suppressed *INV* gene expression. However, the activity of sucrose phosphate synthase (SPS), which is involved in sucrose synthesis, is increased in the cold environment, allowing the soluble sugar content to rise to resist exogenous stress [101]. SWEETs are involved in different stages of pollen development. The accumulation of sugars by SWEETs transporters in low environments contributes to enhanced plant tolerance [102]. AtSWEET16-17 transport fructose and glucose to improve cold tolerance [103]. Although callose is transiently present in pollen, it still affects male reproductive development. Under normal conditions, callose is regularly deposited in the pollen tube wall in a circular form, while under cold stress, callose deposition becomes irregular. Further studies demonstrate that low temperature alters the distribution of callose synthase (CalS), which affects callose deposition [104].

After perceiving external low temperature, plants use phytohormones as signals to mediate the related genes and sugar transporters to affect sugar metabolism and transport, ultimately bringing the growth and reproduction of plants to a new balance under stress conditions. Low temperature increases ABA content by promoting ABA anabolism and inhibiting ABA catabolism. Subsequently, the expression of *OsINV4*, and *OsMST7*-*8* is suppressed, hindering sugar transport into the tapetum and pollen [62], and finally causing pollen abortion. The exogenous application of ABA to anther also had similar results with cold treatment. Cold tolerant plants, however, protect sugar supply and fertility by maintaining lower levels of ABA in anthers. GA has an antagonistic effect with ABA in reverse stress resistance. Cold stress reduces GA content by inhibiting biosynthesis and signal transduction in the tapetum. Sucrose transporters AtSWEET13 and AtSWEET13 transport GA, and GA partially restores pollen dehiscence defects in *atsweet13 atsweet14* double mutant [105]. In conclusion, sugar transport and metabolism and plant hormones, as key factors, are mutually interacted to coordinate anther cold tolerance.

## 7. Conclusions and Perspective

Low temperature impairs the whole process of reproductive development. Tapetum and pollen are the most sensitive to cold. Low temperature forces hypertrophy of the tapeta to delay their PCD, thus affecting nutrient transport from the tapeta to microspores, causing the loss of pollen fertility and activity. As a plant endogenous messenger, low temperature makes ABA levels rise or GA inhibition by affecting the metabolism of ABA or GA in anthers. These changed hormones in turn affect the tapetal PCD, which is prevented by ABA and promoted by GA for its degradation. In addition, the elevated ABA content inhibits transporters and multiple sugar transporters, affecting sugar transport, which makes sugar accumulation insufficient within pollens, leading to sterility. Moreover, GA and sugar are considered as regulators in developmental timing and age pathway, which also positively links with cold stress [106]. We therefore hypothesized that the different developmental process of pollen might be associated with the different cold-tolerance.

Multiple regulatory pathways exist during anther development in response to cold stress. A comparison of anther transcriptomes between cold-tolerant and cold-sensitive cultivars indicates that the differentially expressed genes (DEGs) are included in phytohormone signaling pathways, carbohydrate pathways, the biosynthesis of secondary metabolites, ribosomes, as well as some transcription factor families [90]. Of them, upregulated genes are related to reproductive organ development, while downregulated genes are related to photosynthesis and sugar metabolism [19,107]. Interestingly, the DEGs of the phytohormone signaling pathways include amounts of ABA-, GA-, AUX- and ethylene-related genes. Compared with cold-sensitive plants, cold-tolerant plants can maintain fertility for their lower ABA levels and normal carbohydrate levels in cold environment. Thence, revealing the interaction between different hormones and sugars in mediating cold stress at the anther development process is an important study clue.

Elucidating the mechanism by which upstream signal in response to cold stress in plants is also a future direction [108]. The transcriptome analysis of wheat and rice anthers revealed that the differentially expressed transcription factors under cold mainly belonged to the TF families of MYB, zinc finger, and bZIP [108]. Until now, overexpressing or silencing ABA-, GA- and sugar-related genes, alter the plant cold tolerance in anther. However, how the plants perceive cold signal and deliver it to the reproductive cells, and further regulate these gene expressions against environment, should be intensively studied in the future.

**Figure 2:**
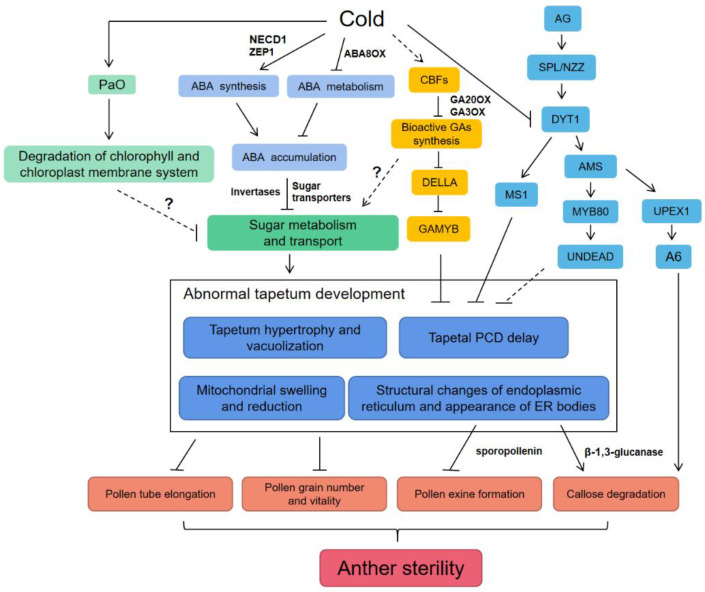
Cold stress disrupts anther fertility by affecting tapetal development. Cold stress affects tapetal PCD by regulating ABA and GA levels. Cold can also affect sugar metabolism and transport, resulting in nutrient deficiencies in the tapetum and microspores within anthers. Hypertrophy and vacuolization of the tapetum in cold environments delayed tapetal PCD and impaired pollen development.

Extreme temperatures affect the reproductive development and yield of crops, so exploring the mechanisms of crops response to cold stress can lead to directions for screening cold-tolerant crops and protect food yield security. The effect of low temperature on meiosis during anther development can produce a diverse genome. Cold stress contributes to the screening of new varieties and species evolution. Herein, we summarized recent research progress on the regulation of the tapetal PCD and pollen sugar metabolism in anthers by ABA and GA at low temperature, ultimately affecting pollen fertility (Figure 2), with the hope of refining the molecular mechanisms underlying pollen responses to cold stress. However, the interrelationship of phytohormones in anther development, as well as the effect of GA on sugar synthesis and transport in pollens, is currently unclear and remains to be explored in the future.
